# Spatial Distribution of Changes in Oxidised Cytochrome C Oxidase During Visual Stimulation Using Broadband Near Infrared Spectroscopy Imaging

**DOI:** 10.1007/978-3-319-38810-6_26

**Published:** 2016-05-02

**Authors:** P. Phan, D. Highton, S. Brigadoi, I. Tachtsidis, M. Smith, C. E. Elwell

**Affiliations:** 10 Department of Medical Physics and Biomedical Engineering, University College London, London, UK; 11 Neurocritical Care, National Hospital for Neurology and Neurosurgery, London, UK; 12 NIHR University College London Hospitals Biomedical Research Centre, London, UK

**Keywords:** Near infrared spectroscopy, Functional imaging, Cytochrome coxidase, Diffuse optical imaging, Visual cortex

## Abstract

Functional hyperaemia, characterised as an increase in concentration of oxyhaemoglobin [HbO_2_] and a decrease in concentration of deoxyhaemoglobin [HHb] in response to neuronal activity, can be precisely mapped using diffuse optical spectroscopy. However, such techniques do not directly measure changes in metabolic activity during neuronal activation. Changes in the redox state of cerebral oxidised cytochrome c oxidase Δ[oxCCO] measured by broadband spectroscopy may be a more specific marker of neuronal metabolic activity. This study aims to investigate the spatial distribution of Δ[oxCCO] responses during the activation of the visual cortex in the healthy adult human brain, and reconstruct images of these changes.

Multi-channel broadband NIRS measurements were collected from the left visual cortex of four healthy volunteers using an in-house broadband spectrometer during an inverting checkerboard visual stimulation paradigm. Δ[HbO_2_], Δ[HHb] and Δ[oxCCO] were calculated by fitting the broadband spectra between 780 and 900 nm using the UCLn algorithm. Centre of gravity analysis was applied to the concentration data to determine the centres of activation for [HbO2], [HHb] and [oxCCO].

All four subjects showed similar changes in [oxCCO] in the presence of a typical visual-evoked haemodynamic response in channels overlying the visual cortex. Image reconstruction of the optical data showed a clear and spatially localized activation for all three chromophores. Centre of gravity analysis showed different localisation of the changes in each of the three chromophores across the visual cortex with the x-y coordinates of the mean centres of gravity (across 4 subjects) of HbO_2_, HHb and oxCCO at (63.1 mm; 24.8 mm), (56.2 mm; 21.0 mm) and (63.7 mm; 23.8 mm), respectively.

The spatial distribution of Δ[oxCCO] response appears distinct from the haemodynamic response in the human visual cortex. Image reconstruction of Δ[oxCCO] shows considerable promise as a technique to visualise regional variation in [oxCCO] in a range of scenarios.

## Introduction

Near-infrared spectroscopy (NIRS) is a Near-infrared spectroscopy (NIRS) that characterises cerebral haemodynamics and metabolism using the attenuation of near infrared light (700–1000 nm) to derive concentration changes in oxyhaemoglobin Δ[HbO_2_], deoxyhaemoglobin Δ[HHb] and the oxidation status of cytochrome c oxidase Δ[oxCCO].

[HbO_2_] and [HHb] are popular targets for HbO2. Several studies using Diffuse optical imaging have been able to produce detailed [HbO_2_] and/or [HHb] maps of various functional regions [1, 2]. However, it is notable that changes in [HbO_2_] and [HHb] provide information only on the haemodynamic responses to neuronal activity and do not inform directly on the changes in cellular metabolism associated with functional activation.

Cytochrome c oxidase is the terminal electron acceptor in the Oxidised cytochrome c oxidase (oxCCO) and directly responsible for more than 95 % of oxygen metabolism [3]. Δ[oxCCO] is therefore a direct and reliable marker of changes in cellular oxygen metabolism, but its measurement requires an optimised broadband spectroscopic technique [4]. There are no published data on the spatial distribution of cytochrome c oxidase or imaging of changes of cytochrome c oxidase redox state across the human Oxidised cytochrome c oxidase (oxCCO). As [oxCCO] is directly related to cellular oxygen metabolism, the ability to measure the spatial distribution of the signal and image these changes may facilitate opportunities to investigate regional cerebral metabolism across a wide range of scenarios.

This study aims to utilise visual stimulation to (1) assess the spatial distribution of [oxCCO] responses resulting from functional brain activation, and (2) evaluate the possibility of reconstructing images of these responses. A visual stimulation paradigm was chosen given its capability to produce a highly repeatable and well-characterised functional activation response that has been corroborated across multiple imaging modalities, including broadband spectroscopy and functional magnetic resonance imaging [1, 5].

## Methods

**Fig. 26.1:**
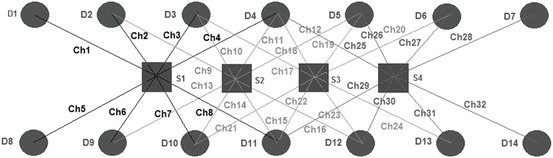
Array of source (*square*) and detector (*circle*) locations. Measurement channels between different source-detector pairs are labelled from Ch1 to Ch32. For example source location S1 resulted in Near-infrared spectroscopy (NIRS) Ch1–Ch8

A detailed description of the Near-infrared spectroscopy (NIRS) used in this study can be found elsewhere [6]. In summary, it utilises a 50 W halogen light source and two CCD cameras (Pixis 512, Princeton Instruments, Trenton, NJ, USA) each connected to four detectors. Figure 26.1 shows the optical array which incorporates four source locations 2 cm apart. This results in source detector Near-infrared spectroscopy (NIRS)s of 2.5 cm and 3.78 cm. The optode positions were digitised using a Patriot™ Patriot( Digitizer (Polhemus, Colchester, Vermont, USA).

Visual functional activation was achieved with a 4Hz inverting checkerboard delivering 20 s stimulation and 20 s black screen repeating over 10 epochs. The optode array was fixed horizontally with the fourth source location positioned over Oz (10/20 EEG position).

Intensity data were continuously collected during the 10 epochs using eight detector fibres arranged symmetrically around one broadband light source. This was repeated for each of the four source locations by translating the fibres over the fixed optode array, yielding an aggregate of 32 measurement channels. Δ[HbO_2_], Δ[HHb] and Δ[oxCCO] were derived using the UCLn algorithm Near-infrared spectroscopy (NIRS) over the wavelength range 780 nm to 900 nm. The wavelength dependence of the differential pathlength factor (DPF) was taken into account when resolving concentration changes, as described by Matcher et al. [4]. The specific requirements for applying this method for measuring changes in chromophore concentrations in the adult head have been described in detail elsewhere [6].

There were 32 time-series datasets of concentration changes for each of the four subjects. Concentration changes were averaged across epochs producing 32 40 s traces corresponding to 20 s of stimulation and 20 s of rest.

Near-infrared spectroscopy (NIRS) [7] was applied on the time-course datasets to investigate the localisation of changes in the three chromophores using the 2.5 cm source-detector separation channels. The location of detector 8 was taken as the origin and all units are in mm. The centres of gravity were determined using the mean response amplitudes of the changes occurring during time *t* = 15–20 s of the each individual stimulation and the coordinates of the channels [7]. Ten epochs produced ten centres of gravity for each chromophore. Mean coordinates of the ten repeats were calculated for the three chromophores to produce the final centres of gravity with 95 % CI.

A Near-infrared spectroscopy (NIRS), which directly reconstructs images of concentration changes from attenuation data, was employed to reconstruct Δ[HbO_2_], Δ[HHb] and Δ[oxCCO]. Data at seventeen wavelengths were selected from the measured broadband spectrum (every 10 nm from 740 to 900 nm) to perform the reconstruction [8]. TOAST++ software [9] was used to run the forward model on the registered adult volumetric mesh and the Tikhonov regularized least-square solution was used to solve the inverse problem. The volumetric images were then projected on the cortical surface mesh.

## Results

**Fig. 26.2:**
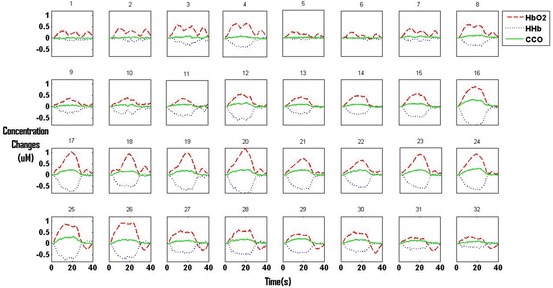
Averaged Δ[HbO_2_], Δ[HHb] and Δ[oxCCO] from a single subject. Stimulation occurred between 0 and 20 s and rest between 20 and 40 Oxidised cytochrome c oxidase (oxCCO) HHb HbO2 Visual cortex

Following ethics approval and volunteer consent, four healthy adults were studied. Figure 26.2 shows the average changes of [HbO_2_], [HHb] and [oxCCO] in all 32 channels over the left Oxidised cytochrome c oxidase (oxCCO) HHb HbO2 Visual cortex of a single subject which is representative of the data acquired from all four subjects. A typical haemodynamic response to functional activation (increase in [HbO_2_] and decrease in [HHb]) was seen across different channels.

**Fig. 26.3:**
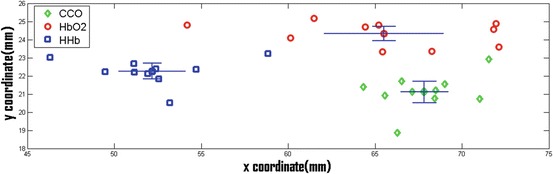
Centres of gravity of the three chromophores for individual repeats. Overall centres of gravity are the intersection between the two 95 % CI lines for each Near-infrared spectroscopy (NIRS)

Figure 26.3 shows the result of Near-infrared spectroscopy (NIRS) for the same subject There is no overlap between 95 % CI in *x* and *y* directions between centres of gravity of [oxCCO] and [HHb] and between [HHb] and [HbO_2_], suggesting that they have distinctly separate locations. In the case of [oxCCO] and [HbO_2_], there is no overlap in the *y* direction but some in the *x* direction, suggesting a less distinct spatial separation than that seen for [HHb]. The centres of gravity for all subjects are summarised in Table 26.1.

**Table 26.1:** Mean *X Y* coordinates ± SD of Oxidised cytochrome c oxidase (oxCCO) HbO2 HHb for three Near-infrared spectroscopy (NIRS) in four subjects

Subject	HbO_2_		HHb		oxCCO	
	*X* (mm)	*Y* (mm)	*X* (mm)	*Y* (mm)	*X* (mm)	*Y* (mm)
1	62.2 ± 5.5	24.4 ± 1.4	59.6 ± 3.6	20.4 ± 1.0	59.7 ± 2.6	25.1 ± 1.6
2	65.5 ± 5.5	24.3 ± 0.6	52.2 ± 3.1	22.3 ± 0.7	67.8 ± 2.2	21.1 ± 1.0
3	62.6 ± 6.4	24.0 ± 1.5	55.4 ± 2.2	21.2 ± 0.9	64.9 ± 2.0	24.1 ± 0.9
4	62.1 ± 4.5	26.6 ± 1.3	57.7 ± 1.3	20.1 ± 0.8	62.5 ± 2.7	24.9 ± 1.5
Mean	63.1 ± 0.8	24.8 ± 0.6	56.2 ± 1.6	21.0 ± 0.5	63.7 ± 1.7	23.8 ± 0.9

**Fig. 26.4:**
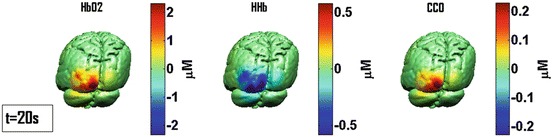
Images of concentration changes of three chromophores for the same subject shown for time point *t* = 20 s demonstrating spatial distribution of Near-infrared spectroscopy (NIRS)

Figure 26.4 shows cortical images of reconstructed concentration changes for the subject shown in Figs. 26.2 and 26.3. Time point *t* = 20 s shows the maximal changes during the stimulation period. These images show different localisations between [HbO_2_]/[oxCCO] and [HHb] changes, whereas a similar region is active for [HbO_2_] and [oxCCO], consistent with the centres of gravity analysis results.

## Discussion

We have demonstrated focal localisation of Δ[oxCCO] that is discrete from the haemodynamic signal ([HbO_2_], [HHb]) during activation of the Visual cortex. The regional separation of changes in each chromophore may reflect the effect of surrounding/overlying vasculature versus regions of direct metabolic activity. The distinct spatial localisation of HbO_2_ and HHb might be explained by the fact that the HbO_2_ signal is derived from arteries, capillaries and veins whereas the HHb signal is derived mostly from HHb within the region of interest and therefore affected by the difference in distribution of arteries and veins in the field of view. Such separation in the centres of gravity for HbO_2_ and HHb has previously been demonstrated by Koenraadt et al. [7]. Furthermore we have illustrated, for the first time, the feasibility of reconstructing Δ[oxCCO] images from a broadband NIRS array. Previous authors have simulated broadband NIRS image reconstruction for [HbO_2_] and [HHb] [10], but in vivo investigation has been limited by the lack of easily accessible and appropriate hardware. We have used an experimental paradigm with a robustly reproducible response and multiple changes in source position to provide multichannel, multiwavelength data required for image reconstruction of [oxCCO], but this approach does have practical limitations. Further work is required to develop optimised hardware to deliver a multispectral NIRS multichannel array capable of real-time recordings. The delivery of Δ[oxCCO] topography and image reconstruction will allow investigation of regional changes in Near-infrared spectroscopy (NIRS) in the healthy and injured brain.
